# Integrating Earth Dynamics Across Dimensions: the next main theme of Earth science

**DOI:** 10.1093/nsr/nwag226

**Published:** 2026-04-16

**Authors:** Zhengtang Guo, Yupeng Yao, Zhe Liu

**Affiliations:** Department of Earth Sciences, National Natural Science Foundation of China, China; Institute of Geology and Geophysics, Chinese Academy of Sciences, China; Department of Earth Sciences, National Natural Science Foundation of China, China; Department of Earth Sciences, National Natural Science Foundation of China, China

The Department of Earth Sciences at the National Natural Science Foundation of China (NSFC) recently announced its key funding priorities for the 15th Five-Year Plan period (https://www.nsfc.gov.cn/p1/2931/3971/3979/4058/dqkxb222222.html) and will begin accepting proposals for key projects in 2026. Building on the priorities established during the 14th Five-Year Plan [1,2] and aligning with the broader vision of Integrating Earth Dynamics Across Dimensions [3], this new plan has drawn considerable attention from the research community. It places greater emphasis on fundamental breakthroughs in Earth science, calls for stronger interdisciplinary collaboration—both within the field and with other disciplines, encourages a paradigm shift in research approaches and stresses the need to better connect basic research with pressing societal challenges.

This strategic direction stems from a deep analysis of trends in Earth science and urgent societal needs. Over the past century or so, the central focus, or ‘main theme’, of Earth science has shifted every 20−30 years. In the early 1900s, breakthroughs such as the glacial cycle theory [4], the discovery of the Mohorovičić discontinuity [5] and the hypothesis of continental drift [6] opened new frontiers in understanding Earth’s environment and internal dynamics. The 1930s–1940s saw the development of the astronomical theory of ice ages, culminating in the Milankovitch theory [7], which became a cornerstone of Earth environmental science. The 1960s–1970s brought plate tectonics [8], which revolutionized the study of Earth’s solid spheres, became a landmark achievement in 20th-century natural science, and greatly advanced research on resources and natural hazards. Then, in the 1980s, the rise of global change research shifted focus to understanding the dynamics of Earth’s surface fluid spheres in response to environmental challenges, delivering unprecedented insights and reshaping perspectives on global economic development.

Today, however, a growing number of scientists recognize that studying Earth’s solid spheres alone is insufficient for solving resource-related problems. Many mineral and resource deposits result from the interactions of tectonic and environmental systems; each can be regarded as a 4D system in motion. Moreover, certain key processes of the solid Earth are themselves shaped by climate and life. Similarly, focusing solely on surface fluid spheres cannot fully address environmental issues, because solid Earth dynamics—through their control over land–sea distribution, topography and deep-to-surface carbon cycling—also regulate climate and environmental change.

Moreover, human activity has become a major force within the Earth system. Although climate changes on geological timescales are generally slow and long-term, whereas societal changes are relatively fast and short-term, it is essential to integrate both perspectives. Environmental outcomes arise from the combination of variabilities across different timescales, each often driven by distinct factors and mechanisms. Changes at societal scales, characterized by small and sometimes subtle variability, reveal only part of the picture. In contrast, geological-scale processes—with their longer timescales and larger, more readily observable variability—act as a ‘magnifying glass’ for uncovering the fundamental laws and mechanisms that underlie shorter-term dynamics.

More importantly, recent studies [9] have highlighted significant cross-scale effects (both downscaling and upscaling) in environmental evolution, further emphasizing the need to integrate processes across different timescales to understand environmental changes relevant to humanity. In other words, without a sound explanation for long-term climate behaviors—such as Cenozoic global cooling, glacial–interglacial cycles and abrupt climate changes—we cannot fully understand how the climate system works, let alone make accurate predictions of future change.

In this context, the Earth science community has begun to emphasize the connections between Earth’s internal dynamics, surface environment, life evolution and human activities [3,10]. Although specific research foci vary, a common scientific goal has emerged: understanding the physical, chemical, biological and geological processes and mechanisms that link all components of the Earth system across timescales—from geological to human. While NASA introduced the term ‘Earth System Science’ in the 1980s [11], and global change research was regarded as a new paradigm—with climate models that incorporate carbon cycles being called ‘Earth System Models’—many argue that the early concept of the ‘Earth system’ referred primarily to the surface system. The new emphasis on dynamic linkages across all Earth spheres and all timescales represents a truly comprehensive Earth system science and poses a new grand challenge. In this sense, the integration of Earth dynamics across dimensions is emerging as the new ‘main theme’ of Earth science, following in the footsteps of plate tectonics and global change research. It holds promise as a breakthrough point for addressing climate, environmental, resource and hazard issues, and may well represent the frontier for the next major theoretical advance in Earth science.

Scientific progress is not a linear accumulation of knowledge; it occurs through paradigm shifts that encompass both concepts and methods. For Earth science, integrating cross-sphere dynamics across time first requires a conceptual shift. Given the substantial progress already made in integrating processes within Earth’s solid and fluid spheres over specific timescales, the next major challenge is to integrate dynamics across dimensions—breaking through spatial and temporal scales and linking the solid, fluid and life spheres [1]. Achieving this conceptual shift urgently calls for a transformation in methodology: moving from single-discipline to truly interdisciplinary research, from qualitative to quantitative process studies and from relatively independent observation and modeling toward a data–model dual-driven research system supported by new data acquisition and big data. This transformation certainly includes the application of artificial intelligence (AI) in Earth sciences, which facilitates a paradigm shift, underpins a new revolution in Earth science and at the same time provides critical application scenarios for advancing AI itself.

Looking at the current state of the field, global-scale atmosphere–ocean models are relatively mature. Due to the complexity of processes and mechanisms, as well as limitations in observation technology and computing power, numerical models for the solid Earth and deep interior remain fragmented. Developing integrated detection technologies and computing capabilities to build global-scale numerical models of the deep and solid Earth is an urgent task. Solving key scientific puzzles, such as the driving mechanisms of plate tectonics, the origin of ice ages and the relationship between life and the environment, depends on such large-scale models. Looking ahead, in the new era of integrating Earth dynamics across dimensions, nations that truly establish a data–model dual-driven research system—pioneering advanced numerical models that fully couple Earth’s solid, fluid and human spheres, supported by new data and big data—are likely to become leaders in future Earth science and major contributors to the next major theory. Building such a research system is undoubtedly a long-term goal of great significance, representing a true scientific commanding height.

The 10 key funding priorities outlined by the NSFC Department of Earth Sciences for the 15th Five-Year Plan are precisely designed to embrace this new ‘main theme’ of Earth science. They include: (1) carbon and water cycles in the Earth system; (2) global change and nature–society coupling; (3) Earth and planetary habitability and the origin of life; (4) human civilization evolution and the environment; (5) cross-sphere material cycling: resources and environment; (6) ocean system evolution and cross-sphere feedbacks; (7) emerging pollutant cycling and control; (8) cross-sphere disaster dynamics and prevention; (9) Earth system modeling and AI; and (10) new observation methods and technologies for integrating cross-sphere dynamics. Priorities 1–4 target scientific frontiers, 5–8 are more oriented toward societal needs, and 9 and 10 focus on building a data–model dual-driven research system. This funding framework also emphasizes the cultivation of interdisciplinary talent in Earth science. It is hoped that research in these cross-cutting areas will help foster a ‘new school’ of thought in global Earth science, positioning China as a leader in the field and a major contributor to the next theoretical breakthrough, while addressing critical knowledge needs related to resources, the environment and natural hazards for both China and the world.
